# Acute Kidney Injury and 3-Year Mortality in Elderly Patients After Non-cardiac Surgery

**DOI:** 10.3389/fmed.2022.779754

**Published:** 2022-04-12

**Authors:** Qiong-Fang Wu, Mao-Wei Xing, Wen-Jun Hu, Xian Su, Dan-Feng Zhang, Dong-Liang Mu, Dong-Xin Wang

**Affiliations:** ^1^Department of Anesthesiology and Critical Care Medicine, Peking University First Hospital, Beijing, China; ^2^Department of Anesthesiology, The 305th Hospital of the Chinese People's Liberation Army, Beijing, China; ^3^Department of Anesthesiology, Fujian Medical University Union Hospital, Beijing, China

**Keywords:** acute kidney injury, non-cardiac surgery, 3-year, survival, the elderly

## Abstract

**Objective:**

The present study aimed to investigate whether acute kidney injury (AKI) was associated with 3-year mortality in elderly patients after non-cardiac surgery.

**Methods:**

The present study was a 3-year follow-up study of two randomized controlled trials. A total of 1,319 elderly patients who received non-cardiac surgery under general anesthesia were screened. AKI was diagnosed by the elevation of serum creatinine within a 7-day postoperative period according to Kidney Disease: Improving Global Outcomes (KDIGO) guidelines. A long-term telephonic follow-up was undertaken by investigators who were not involved in the previous two trials and had no access to the study group assignment. The date of death was taken from the official medical death certificate. The primary outcome was to investigate the association between AKI and postoperative 3-year mortality using the multivariable Cox regression risk model.

**Results:**

Of the 1,297 elderly patients (mean age 71.8 ± 7.2 years old) who were included in the study, the incidence of AKI was 15.5% (201/1297). Of the patients with AKI, 85% (170/201) were at stage 1, 10% (20/201) at stage 2, and 5% (11/201) at stage 3. The 3-year all-cause mortality was 28.9% (58/201) in patients with AKI and 24.0% (263/1,096) in patients without AKI (hazard ratio 1.247, 95% confidence interval 0.939–1.657, *P* = 0.128). The multivariable Cox regression showed that AKI was not associated with 3-year mortality after adjustment of confounding factors (adjusted hazard ratio 1.045, 95% confidence interval 0.780–1.401, *P* = 0.766).

**Conclusions:**

AKI was a common postoperative complication, but it was not associated with 3-year mortality in elderly patients who underwent non-cardiac surgery. The low incidence of severe AKI might underestimate its underlying association with long-term mortality.

## Introduction

Postoperative mortality is the third largest contributor to global death, following ischemic heart disease and stroke (1, 2). Complications such as major adverse cardiac events, stroke, and acute kidney injury are considered as the main reasons for perioperative death (3–5). Among these complications, acute kidney injury (AKI) presents a higher incidence and has a significantly detrimental effect on clinical outcomes.

The overall incidence of AKI varies from 3 to 13% in patients who underwent non-cardiac surgeries and increases up to 20% in older adults (5–7). The underlying etiologies and mechanisms of AKI are multifactorial. Common risk factors include poor renal function (i.e., chronic renal disease), surgery-related stress response, nephrotoxic drugs (i.e., anesthetics and antibiotics), and hemodynamic variability (i.e., hypotension and hypovolemia) (6, 8, 9).

Acute kidney injury is associated with poor clinical outcomes in patients. Several studies indicated that patients with AKI suffer three times the risk of cardiovascular complications and prolonged in-hospital stay (9–11). In a population-based study, patients with AKI experienced an 8-fold increased risk of 30-day mortality (12). Patients with AKI, after vascular surgery, spent an additional amount of US$ 9,000–19,000 on confounding comorbidities and other postoperative complications (13).

Unlike the supportive data on AKI and postoperative short-term (i.e., within 30 days) mortality, it is still uncertain whether AKI is associated with long-term mortality in patients after non-cardiac surgery. After an abdominal surgery, the risk of 1-year mortality increased by 2-fold in patients with AKI (14). This result is consistent with a meta-analysis of 82 cohort studies which showed that patients with AKI were at an increased risk of long-term mortality (13.19 vs. 7.26 deaths per 100 person-years) (15). However, a recent observational study did not find the impact of AKI on 1-year mortality in patients after colorectal cancer surgery (16). A prospective study of critically ill patients also found that AKI was not an independent risk factor of 3-year mortality (17). Evidence from previous studies was mainly based on retrospective designs with inherent flaws, such as missing data and bias (14–16). Strong evidence mapping the relationship between AKI and 3-year mortality in elderly patients after non-cardiac surgery is still lacking (14–18). We hypothesized that patients with AKI may have a higher 3-year mortality compared to patients without AKI after non-cardiac surgery.

The present study was designed to investigate whether AKI was associated with 3-year mortality in elderly patients after non-cardiac surgery.

## Materials and Methods

This analysis was based on a 3-year follow-up dataset of two randomized controlled trials (RCTs) (19, 20). The study protocol was approved by the Clinical Research Ethics Committee of Peking University First Hospital (2020-115). Informed consents were waived because of no intervention in patients.

### Participants

Participants in two RCTs were prospectively followed up for 3 years (Supplementary File 1). In the first trial, elderly patients aged 65 and above were enrolled from 17 August 2011 to 20 November 2013 to investigate the primary hypothesis, that is, if a low dose intraoperative infusion of dexmedetomidine for sedation could decrease the incidence of postoperative delirium in intensive care unit (ICU) patients (19).

The two trials had similar exclusion criteria: 1) previous history of schizophrenia, epilepsy, or Parkinson's disease; 2) inability to communicate; 3) history of traumatic brain injury or neurosurgery; 4) severe bradycardia (heart rate <50 beats per minute), sick sinus syndrome or atrioventricular block of degree 2 or above without pacemaker; 5) severe hepatic dysfunction (Child-Pugh grade C), and 6) renal failure or requirement for renal replacement therapy before surgery.

In the present study, we excluded patients who had no serum creatinine at baseline or within a 7-day postoperative period and patients who underwent renal transplantation surgery.

### Definition of AKI

The criteria of Kidney Disease: Improving Global Outcomes (KDIGO) was used to diagnose AKI (21). As the value of urine output (i.e., per hour or per 6 h) could not be accurately collected from patients in the general ward, we utilized the variability in serum creatinine measurements to diagnose AKI in accordance with most clinical studies (5, 6).

In comparison with the baseline value, AKI was established when there was an increase in serum creatinine by ≥ 0.3 mg/dl within 48 h after surgery or an increase in serum creatinine of ≥ 1.5 times baseline within a 7-day postoperative period. The severity of AKI was divided into stage 1 (postoperative creatinine was 1.5–1.9 times baseline or increased by ≥ 0.3 mg/dl within 48 h after surgery), stage 2 (postoperative creatinine was 2–2.9 times baseline), and stage 3 (postoperative creatinine was > 3 times baseline or increased by ≥ 4.0 mg/dl or the commencement of renal replacement therapy) respectively (21).

Depending on when it occurred, AKI within a 48-h postoperative period was defined as early AKI, and AKI occurring from 48 h to 7 days after surgery was termed late AKIn (22).

### Follow-Up and Definition of Survival

The 3-year follow-up was performed by investigators who had not been involved in the previous two trials and had no access to study group assignments. Follow-up was performed by telephonic interviews with patients and/or their family members. For patients who died within 3 years, the date of death was recorded as per the official medical death certificate. A telephonic interview was tried at least 5 times on 5 different days before patients were marked as lost to follow-up. For these patients, the time of their last hospital visit after surgery as recorded in the inpatient or outpatient medical record system was defined as censoring time.

### Outcomes

The primary outcome was to investigate the association between AKI and 3-year mortality. The secondary outcomes were the associations between the onset time and the severity of AKI and 3-year mortality.

### Data Collection

Demographic characteristics, preoperative comorbidities, the American Society of Anesthesiologist (ASA) classification, surgery and anesthesia-related information, non-AKI complications within a 30-day postoperative period and perioperative serum creatinine levels were collected from the database of the in-hospital electronic medical system and case report forms. The 3-year follow-up data, including survival time and the cause of mortality, was collected through telephonic interviews.

### Statistical Analysis

Prior to analyzing the data, the normality of continuous data was tested using the Kolmogorov-Smirnov test. Data with normality were expressed as mean ± standard deviation (SD), and the between-group difference was compared through the independent sample *t-test*. Data without normality were expressed as median (interquartile range, IQR), and the between-group difference was compared using the independent sample Mann-Whitney U test. Categorical variables were expressed as numbers (percentage) and compared using the chi-square or Fisher's test.

For the primary outcome, the Kaplan-Meier analysis was used to explore the relationship between AKI and 3-year mortality. Comparison between the two groups was tested by the log-rank test. A univariate analysis was used to screen potential risk factors that might be associated with 3-year mortality (i.e., baseline characteristics, perioperative variables, and postoperative non-AKI complications). Variables with *P* < 0.05 in the univariate analysis or that were considered clinically important (i.e., perioperative dexmedetomidine infusion) were included in a multivariable Cox regression analysis. AKI was compulsorily entered into the multivariable Cox regression analysis. The relationships between the onset time and the severity of AKI and 3-year mortality were also analyzed similarly.

Statistical analysis was conducted by SPSS 25.0 (SPSS, Inc., Chicago, IL). All tests were two-tailed, and *P* < 0.05 was considered as statistical significance.

## Results

### Patients

**Figure 1:**
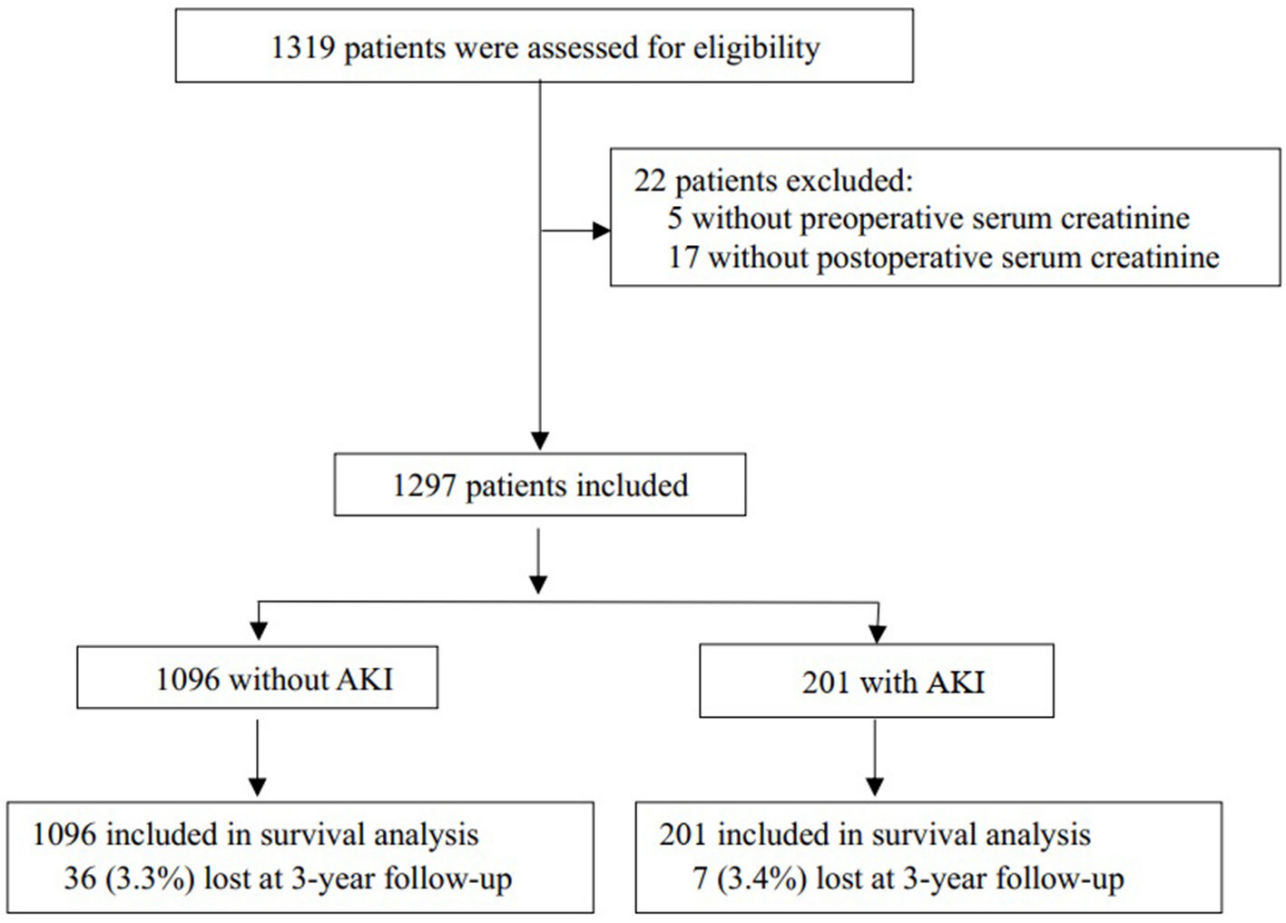
A flowchart of the study. AKI, acute kidney injury.

A total of 1,319 patients were eligible, of which 1,297 patients entered the final analysis (Figure 1). The mean age of the enrolled patients was 71.8 ± 7.2 years. The median follow-up time was ~36 (IQR 30, 42) months. Baseline characteristics and perioperative variables are listed in Tables 1, 2.

**Table 1:** Demographic and baseline variables.

	**All patients (*n* = 1,297)**	**Non-AKI group (*n* = 1,096)**	**AKI group (*n* = 201)**	***P-*value**
Age, yr	71.8 ± 7.2	71.6 ± 7.2	73.3 ± 7.1	0.001
Sex (male), *n* (%)	778 (60.0)	665 (60.7)	113 (56.2)	0.24
Body mass index, kg·m^−2^	23.9 ±3.6	23.8 ± 3.5	24.3 ± 4.1	0.08
Body mass index < 18.5 kg·m^−2^	89 (6.9)	74 (6.8)	15 (7.5)	0.71
Preoperative comorbidities, *n* (%)				
Stroke	220 (17)	176 (16.1)	44 (21.9)	0.04
Hypertension	723 (55.7)	595 (54.3)	128 (63.7)	0.01
Diabetes	318 (24.5)	259 (23.6)	59 (29.4)	0.08
Cardiac ischemic disease	320 (24.7)	253 (23.1)	67 (33.3)	0.002
Chronic obstructive pulmonary disease	50 (3.9)	37 (3.4)	13 (6.5)	0.04
Malignant tumor ^*^	1,037 (80)	870 (79.4)	167 (83.1)	0.23
History of smoking	318 (25.1)	281 (25.6)	46 (22.2)	0.41
Serum creatinine, mg/dl	1.0 (0.8, 1.1)	1.0 (0.8, 1.1)	1.0 (0.8, 1.2)	0.08
> 1.2 mg/dl, *n* (%)	173 (13.3)	126 (11.5)	47 (23.4)	<0.001
ASA classification, *n* (%)				
I	77 (5.9)	65 (5.9)	12 (6.0)	<0.001
II	860 (66.3)	751 (68.5)	109 (54.2)	
III	360 (27.8)	280 (25.5)	80 (39.8)	
CCI, score	4 (2,4)	4 (2,4)	4 (2,4)	0.87

*AKI, acute kidney injury; SD, standard deviation; ASA, American Society of Anesthesiologists; CCI, Charlson's comorbidity index*.

*Data are presented as mean ± SD, median (interquartile range) or number (%)*.

* *Patients who underwent surgery due to the malignant tumor*.

**Table 2:** Perioperative variables.

	**All patients (*n* = 1,297)**	**Non-AKI group (*n* = 1,096)**	**AKI group (*n* = 201)**	***P*-value**
Type of surgery, *n* (%)				0.10
Intra-abdominal	891 (68.7)	743 (67.8)	148 (73.6)	
Intrathoracic	222 (17.1)	191 (17.4)	31 (15.4)	
Spinal and extremital	119 (9.2)	109 (9.9)	10 (5.0)	
Superficial and transurethral	65 (5.0)	53 (4.8)	12 (6.0)	
Type of anesthesia, *n* (%)				0.94
General anesthesia	1218 (93.9)	1029 (93.9)	189 (94.0)	
Combined epidural-general anesthesia	79 (6.1)	67 (6.1)	12 (6.0)	
Perioperative dexmedetomidine, *n* (%) ^*^	656 (50.6)	559 (51.0)	97 (48.3)	0.47
Duration of anesthesia, h	4.7 (3.5, 6.2)	4.8 (3.6, 4.3)	3.8 (3.0, 5.6)	<0.001
Duration of surgery, h	3.3 (2.2, 4.8)	3.5 (2.3, 4.8)	2.6 (1.8, 4.3)	<0.001
Duration of surgery ≥ 2 h, *n* (%)	1,045 (80.6)	908 (82.8)	137 (68.2)	<0.001
Total fluid infusion, l	2.4 (1.6, 3.5)	2.5 (1.6, 3.6)	2.1 (1.5, 3.1)	<0.001
Intraoperative blood transfusion, *n* (%)	172 (13.3)	143 (13.0)	29 (14.4)	0.60
LOS after surgery, d	9 (6,14)	10 (7,14)	7 (5,14)	<0.001
Postoperative non-AKI complications, *n* (%)	272 (21)	226 (20.6)	46 (22.9)	0.47
Delirium^†^	156 (12.0)	128 (11.7)	28 (13.9)	0.37
Stroke	4 (0.3)	0 (0.4)	0 (0.0)	>0.99
Cardiac complications^‡^	59 (4.5)	44 (4.0)	15 (7.5)	0.03
Pulmonary complications^§^	27 (2.1)	19 (1.7)	8 (4.0)	0.06
Sepsis	17 (1.3)	12 (1.1)	5 (2.5)	0.17
Other complications ^**^	70 (5.4)	57 (5.2)	13 (6.5)	0.47
Death within postoperative 30-day, *n* (%)	7 (0.5)	4 (0.4)	3 (1.5)	0.08

*AKI, acute kidney injury; ICU, intensive care unit; h, hour; IQR, interquartile range; LOS, length of in-hospital stay*.

*Data are presented as number (%) or median (interquartile range)*.

* *Dexmedetomidine infusion during surgery or within 24 h after surgery*;

† *Delirium was diagnosed within 7 days after surgery*;

‡ *Cardiac complications included acute coronary syndrome and heart failure*;

§ *Pulmonary complications included pneumonia and respiratory failure*;

** *Other complications included deep venous thrombosis (6); surgical site infection (20); surgical bleeding (22); anastomotic leakage (12); intestinal obstruction (17)*.

### Incidence of AKI

The incidence of AKI was ~15.5% (201/1297). Of the patients with AKI, 85% (170/201) were at stage 1, 10% (20/201) at stage 2, and 5% (11/201) at stage 3. Of these patients with AKI, 5% (10/201) of them received continuous renal replacement treatment during hospitalization. A significant portion of AKI (95%, 191/201) occurred within a 48-h postoperative period and 5% (10/201) of AKI occurred between 48 h and 7 days after surgery.

### AKI and 3-Year Mortality

**Figure 2:**
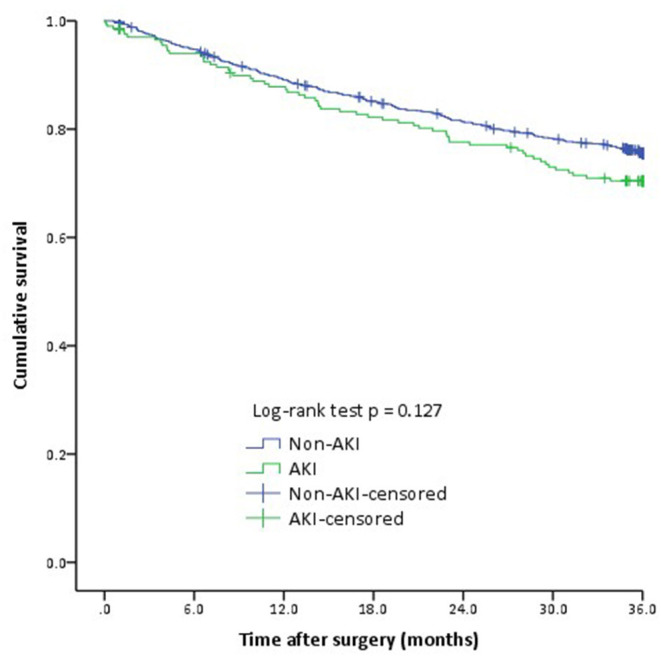
The Kaplan-Meier analysis of 3-year mortality between two groups. Cumulative 3-year mortality was comparable between two groups (Log-rank test *P* = 0.13). AKI, Acute kidney injury.

In total, 24.7% (321/1,297) patients died within 36 months after surgery. The incidence of death was 27.2% (46/170), 35% (7/20), and 45.5% (5/11) in patients with AKI stages 1, 2, and 3, respectively. Overall, the mortality rate was 28.9% (58/201) in patients with AKI and 24.0% (263/1,096) in non-AKI patients (HR 1.247, 95% CI 0.939–1.657, *P* = 0.13). The comparable 3-year mortality rates between patients with and without AKI was done using the Kaplan-Meier analysis (Log-rank test *P* = 0.13, Figure 2).

Furthermore, the multivariable Cox regression analysis showed that AKI was not associated with 3-year mortality (adjusted HR 1.045, 95% CI 0.780–1.401, *P* = 0.77) after adjusting for confounders (i.e., age, gender, BMI <18.5 kg/m^2^, presence of malignant tumors, preoperative serum creatinine > 1.2 mg/dl, ASA classification, major surgery, intraoperative blood transfusion, perioperative dexmedetomidine infusion, and non-AKI complications; Table 3).

**Table 3:** Association between acute kidney injury and 3-year mortality.

**Variables**	**Univariate analysis**		Multivariable Cox regression^*^	
	**HR (95% CI)**	***P*-value**	**HR (95% CI)**	***P-*value**
Age (per 10 years increase)	1.404 (1.210–1.629)	<0.001	1.315 (1.120–1.545)	0.001
Male (yes)	1.384 (1.098–1.744)	0.006	1.399 (1.108–1.766)	0.005
Body mass index < 18.5 kg/m^2^ (yes)	1.809 (1.262–2.593)	0.001	1.614 (1.117–2.332)	0.011
Malignant tumor (yes)	2.689 (1.846–3.916)	<0.001	2.619 (1.784–3.846)	<0.001
Preoperative serum creatinine > 1.2 mg/dl (yes)	1.868 (1.420–2.456)	<0.001	1.512 (1.141–2.004)	0.004
ASA classification (per grade increase)	1.554 (1.269–1.902)	<0.001	1.396 (1.130–1.722)	0.002
Major surgery (surgery time ≥ 2 h, yes)	1.946 (1.387–2.730)	<0.001	1.496 (1.050–2.133)	0.03
Intraoperative blood transfusion (yes)	2.866 (2.232–3.681)	<0.001	2.286 (1.761–2.968)	<0.001
Perioperative dexmedetomidine infusion (yes)	0.868 (0.697–1.080)	0.20	—	—
Acute kidney injury (yes)	1.247 (0.939–1.657)	0.13	1.045 (0.780–1.401)	0.77
Non-AKI complications within 30 days of the postoperative period (yes)	1.537 (1.203–1.964)	0.001	—	—

*HR, hazard ratio; CI, confidence interval; ASA, American Society of Anesthesiologists; AKI, acute kidney injury*.

* *Variables with P < 0.05 in the univariate analysis or considered clinically important (i.e., perioperative dexmedetomidine infusion, acute kidney injury) were included into the multivariable Cox regression analysis*.

### AKI Onset Time and 3-Year Mortality

With regard to patients without AKI, both univariate and multivariable Cox regression analyses showed that late AKI was associated with an increased risk of mortality (adjusted HR 3.754, 95% CI 1.532–9.198, *P* = 0.004; Table 4).

**Table 4:** Association between onset time and stage of acute kidney injury and 3-year mortality.

**Variable**	**Univariate analysis**		Multivariable Cox regression ^*^	
	**HR (95% CI)**	***P*-value**	**HR (95% CI)**	***P*-value**
AKI onset time				
Non-AKI	Reference		Reference	
Early AKI	1.183 (0.881–1.589)	0.264	0.978 (0.722–1.324)	0.88
Late AKI	2.931 (1.210–7.012)	0.017	3.754 (1.532–9.198)	0.004
AKI stage				
Non-AKI	Reference		Reference	
Stage 1	1.133 (0.828–1.550)	0.434	0.958 (0.696–1.320)	0.79
Stage 2 and 3	2.033 (1.140–3.626)	0.016	1.628 (0.903–2.937)	0.11

*HR, hazard ratio; CI, confidence interval; AKI, acute kidney injury*.

* *The multivariable Cox regression model was adjusted for the following confounders: age, gender, BMI < 18.5 kg/m^2^, malignant tumor, preoperative serum creatinine > 1.2 mg/dl, ASA classification, major surgery, intraoperative blood transfusion, perioperative dexmedetomidine infusion, and occurrence of 30-day postoperative non-AKI complications (these factors were listed in Table 3)*.

### AKI Stage and 3-Year Mortality

In patients without AKI, the univariate analysis, but not the multivariable Cox regression analysis, showed that a higher AKI stage (stage 2 and 3) was associated with increased 3-year mortality (HR 2.033, 95% CI 1.140–3.626, *P* = 0.02; Table 4).

## Discussion

The present study found that AKI was not associated with 3-year mortality in elderly patients after non-cardiac surgery. One strength of the present study is the relatively large sample size of older adults. Another strength is that the 3-year follow-up was planned at the beginning of the two trials and all data were prospectively collected.

In the present study, the incidence of AKI was ~15.5%, and 85% of them were at stage 1. This result was consistent with previous studies (5–7). Similar to most other studies, we only employed creatinine to diagnose AKI, and this might underestimate the incidence and the severity of AKI (5, 6).

Many studies have reported the positive relationship between AKI and short-term mortality in patients who underwent non-cardiac surgery, but the association between AKI and long-term mortality is still uncertain (10, 23). A retrospective study analyzed 1,869 patients who underwent non-cardiac surgery and found that AKI was associated with an increased risk of mortality between 8 and 365 days (5). In 6,590 patients who underwent colorectal cancer surgery, AKI was associated with an increased risk of mortality between 8 and 30 days and between 31 and 90 days, but it was not related to mortality between 91 and 360 days (16).

The present study was designed to investigate whether AKI was associated with 3-year mortality in elderly patients after non-cardiac surgery, and our results showed that it was not. This result might be attributed to several underlying factors. First, the impaired kidney function of patients with AKI may recover or improve with time, and this potentially reduces its impact on long-term mortality. A long-term follow-up of 1,893 patients after partial nephrectomy showed that the incidence of AKI within a 7-day postoperative period was about 20% (388/1,893), and the renal function of most of these patients recovered to 90% of baseline function within a year after surgery (24). A propensity score matching study showed that the recovery of the renal function was associated with a decreased risk of 1-year mortality in surgical patients (25). Second, about 80% of these patients received surgery for malignant tumor, and 73.8% of mortalities were related to cancer. The recurrence of tumor and metastasis might be the major reason for long-term mortality and surpasses the potential effect of AKI. Third, the incidence and severity of AKI might be underestimated because we only considered serum creatinine levels to diagnose AKI without urine output.

The stage and the onset time of AKI may also exert different effects on long-term mortality. The relationship between the AKI stage and long-term mortality had been reported in many studies (5, 16, 26). However, we did not find the statistical significance between the AKI stage and 3-year mortality. This might be attributed to the lower occurrence of severe AKI (only 31 patients experienced stage 2 and stage 3 AKI) in the present study. The sample size in our study was not enough to explore the relationship between AKI stage and 3-year mortality. A retrospective study showed that both early and late onset of AKI were related to an increased risk of 1-year mortality in critically ill patients after a major non-cardiac surgery (22). We noticed that late onset AKI, but not early onset AKI, was related to an increased risk of 3-year mortality in the present study. However, only 10 patients were diagnosed with late onset AKI in our study, and this small sample size yielded insufficient statistical power. Thus, this result should be considered as an exploratory analysis and interpreted with caution.

The etiologies of overall mortality are multifactorial. Aging was the robust predictor of mortality in many studies (27). In the validation study of a national risk prediction model for perioperative mortality in non-cardiac surgery, male patients were associated with an increased risk of mortality and were considered as one of the key factors to improve the predictive performance of the risk model (27). Lower BMI was an important manifestation of malnutrition and was highly associated with an increased risk of complications and mortality (28). Preoperative renal function impairment and the ASA physical status classification were reflections of disease severity and, therefore, were validated as confounders of mortality in many studies (29, 30). Major surgery was defined as a surgery duration of more than 2 h, and the prolonged duration of surgery was considered as a risk factor of mortality in patients after the multivariate analysis (14). The role of allogenic blood transfusion in long-term mortality received more attention, but the conclusions remain controversial (31). Although several studies reported that perioperative application of dexmedetomidine might decrease the risk of long-term mortality in surgical patients, the results were inconclusive (32, 33).

The present study has several limitations. First, we only adopted serum creatinine levels for the diagnosis of AKI, and this might underestimate its incidence. Second, this was a prospective follow-up of two RCTs, which excluded some high-risk patients with comorbidities (i.e., severe hepatic or renal diseases). This might limit the generality of our results. Third, we did not get the serum creatinine levels during the 3-year postoperative period or at follow-up, which impeded the assessment of renal function at the 3-year postoperative period. Fourth, only 11 patients were diagnosed as AKI stage 3 in the present study. Our result showed that the incidence of death increased from 27.2 to 45.5% in patients with AKI stages 1 and 3. However, the low incidence of severe AKI might underestimate its underlying association with long-term mortality.

## Conclusion

The present study found that AKI was common in patients after non-cardiac surgery; however, it was not associated with 3-year mortality. The incidence of severe AKI (i.e., stage 3) was very low, and this might underestimate the underlying association between AKI and long-term mortality. Further studies with a large sample size are needed to verify our result.

## Data Availability Statement

The original contributions presented in the study are included in the article/Supplementary Material, further inquiries can be directed to the corresponding author.

## Ethics Statement

The studies involving human participants were reviewed and approved by the Clinical Research Ethics Committee of Peking University First Hospital. The Ethics Committee waived the requirement of written informed consent for participation.

## Author Contributions

Q-FW helped in data acquisition, data analysis, and manuscript drafting. M-WX, W-JH, XS, and D-FZ helped in data acquisition. D-LM helped with conceptual idea and design, data analysis, data interpretation, manuscript drafting, critical revision of the manuscript for the important intellectual content, and supervision. D-XW helped with conceptual idea and design, administrative or material support, and critical revision of the manuscript for the important intellectual content. All authors contributed to the article and approved the submitted version.

## Funding

This trial was supported by the National Key R&D Program of China (Grant No. 2018YFC2001800).

## Conflict of Interest

The authors declare that the research was conducted in the absence of any commercial or financial relationships that could be construed as a potential conflict of interest.

## Publisher's Note

All claims expressed in this article are solely those of the authors and do not necessarily represent those of their affiliated organizations, or those of the publisher, the editors and the reviewers. Any product that may be evaluated in this article, or claim that may be made by its manufacturer, is not guaranteed or endorsed by the publisher.
