# Supplementary material for: Acute Kidney Injury and 3-Year Mortality in Elderly Patients After Non-cardiac Surgery
Source: Front Med (Lausanne). 2022 Apr 12;9:779754. doi: 10.3389/fmed.2022.779754 (PMC9039224; doi:10.3389/fmed.2022.779754)
Supplement: Supplementary file 1 [file Table_1.docx]

**Supplemental file 1 Brief description of two trials**

|  | **Design** | **Inclusion criteria** | **Exclusion criteria** | **Sample size** | **Primary outcome** |
| --- | --- | --- | --- | --- | --- |
| **First Trial *** | RCT | - Age ≥ 60 years - Scheduled to undergo elective major non-cardiac surgery - Expected surgery duration ≥ 2 hours - General anesthesia | - Did not provide written informed consents - History of schizophrenia, epilepsy or Parkinson’s disease - Visual, hearing, language or other barrier that impeded communication and preoperative delirium assessment - History of traumatic brain injury or neurosurgery - Severe bradycardia (HR less than 40 bpm), SSS, or AVB of degree 2 or above without pacemaker - Severe hepatic dysfunction (Child-Pugh grade C) - Renal failure (requirement of renal replacement therapy) - Neurosurgery | 620 | Delirium |
| **Second Trial #** | RCT | - Aged ≥ 65 years - Underwent elective non-cardiac surgery under general anesthesia - Admitted to the ICU after surgery before 2000h | - History of schizophrenia, epilepsy, Parkinsonism, or MG - Inability to communicate in the preoperative period (coma, profound dementia, or language barrier) - Brain injury or neurosurgery - Preoperative LVEF less than 30%, SSS, severe bradycardia (<50 bpm), AVB of degree 2 or above without pacemaker - Serious hepatic dysfunction (Child-Pugh class C) - Serious renal dysfunction (dialysis before surgery) - Low likelihood of survival for more than 24 h | 700 | Delirium |

HR= heart rate; bpm= beats per minute; SSS= sick sinus syndrome; AVB= atrioventricular block; MG= myasthenia gravis; LVEF= left ventricular ejection fractio

***** The first trial was published in British Journal of Surgery (Br J Surg. 2020 Jan;107(2): e123-e132.).

**#** The second trial was published in Lancet (Lancet. 2016 Oct 15;388(10054):1893-1902.).
